# The Physical Demands of the Tree (Vriksasana) and One-Leg Balance (Utthita Hasta Padangusthasana) Poses Performed by Seniors: A Biomechanical Examination

**DOI:** 10.1155/2012/971896

**Published:** 2012-08-29

**Authors:** Sean S.-Y. Yu, Man-Ying Wang, Sachithra Samarawickrame, Rami Hashish, Leslie Kazadi, Gail A. Greendale, George J. Salem

**Affiliations:** ^1^Division of Biokinesiology and Physical Therapy, University of Southern California (USC), 1540 E. Alcazar Street, Los Angeles, CA 90033, USA; ^2^Division of Geriatrics, Geffen School of Medicine at the University of California, Los Angeles (UCLA), 924 Westwood Boulevard, Suite 200, Los Angeles, CA 90024, USA

## Abstract

Yoga is considered especially suitable for seniors because poses can be modified to accommodate practitioners' capabilities and limitations. In this study, biomechanical assessments on healthy seniors (*n* = 20; 70.1 ± 3.8 yr) were used to quantify the physical demands, (net joint moments of force [JMOFs] and muscular activation in the lower extremities) associated with the performance of 3 variations (introductory, intermediate, advanced) of 2 classical Hatha yoga poses – Tree and One-Leg Balance (OLB). ANOVA and Cohen's-*d* were used to contrast the postural variations statistically. The advanced (single-limb, without additional support) versions were hypothesized to generate the greatest demands, followed by the intermediate (single-limb [Tree] and bilateral-limb [OLB] with support) and introductory (bilateral-limb) versions. Our findings, however, suggest that common, long-held conceptions about pose modifications can be counter-intuitive. There was no difference between the intermediate and advanced Tree variations regarding hip and knee JMOFs in both the sagittal and frontal planes (*P* = 0.13–0.98). Similarly, OLB introductory and intermediate variations induced sagittal JMOFs that were in the opposite direction of the classic advanced pose version at the hip and knee (*P* < .001; *d* = 0.98–2.36). These biomechanical insights provide evidence that may be used by instructors, clinicians and therapists when selecting pose modifications for their yoga participants.

## 1. Introduction

Yoga is a popular exercise activity for older adults, with senior participation in the US estimated at greater than 900,000 participants—an increase of 90% between years 2007 to 2010 [1]. Likely contributing to this rise in participation are anecdotal and lay-journal reports that yoga increases joint range of motion, strength, and balance, improves emotional and spiritual wellness, and that it is relatively safe. Moreover, yoga appears to be especially suited for seniors because programs and individual postures can be modified and tailored to accommodate a variety of participant physical capabilities and limitations [2–4]. Unfortunately, well-designed, randomized controlled trials examining yoga in seniors are rare, and little is known regarding the physical demands, program efficacy, and safety of programs designed for older adult participants. Moreover, seniors have diminished strength and balance, reduced joint range of motion, and a greater prevalence of spinal-canal stenosis, osteoarthritis, and hyperkyphosis [5–7]; therefore, yoga programs designed without using evidenced-based prescription may place elders at risk for musculoskeletal and neurological pain and injury (e.g., strains, sprains, and impingements). 

In the present study, we provide some of this evidence by characterizing the physical demands associated with the performance of 3 common variations (we termed these introductory, intermediate, and advanced) of 2 classical Hatha poses, Tree (Vriksasana) and One-Leg Balance (Utthita Hasta Padangusthasana; OLB). The physical demands of yoga can be quantified by estimating the net joint moments of force (JMOFs) and muscular activation patterns generated during performance of the individual poses and their modifications. While performing a pose, external reaction forces (e.g., ground reaction forces; GRFs) acting on body segments produce external JMOFs about the joints. Acting in the opposite direction, these external JMOFs must be met by internal JMOFs generated via muscular and ligamentous constraints in order to maintain the position of the body's center of mass and/or prevent collapse of the limbs. Although increased muscle loading due to JMOFs may stimulate beneficial adaptational responses (e.g., strength and endurance), excessively high JMOFs may lead to the detrimental loading of articular, ligamentous, and capsular structures and exacerbate existing joint pathology (e.g., osteoarthritis; OA) [8, 9]. For example, a pose that induces a high medial (abductor) JMOF at the knee is likely to increase the stress on the medial collateral ligament and raise the joint reaction forces across the lateral condyles. Conversely, high lateral knee (adductor) JMOFs, produced during functional activities (e.g., walking), have been shown to predict OA progression [10, 11]. 

Information regarding the JMOFs and muscle activation patterns associated with yoga poses may be used by instructors, clinicians, and therapists to select the most appropriate pose versions, targeted at each participant's experience, physical capabilities, and injury history. This information may also be used to determine when it is appropriate to “advance” participants by prescribing the more demanding pose variations. For the present investigation, we hypothesized that for, both the Tree and OLB poses, the “introductory” versions (with bilateral-limb and wall support (Tree); bilateral-limb and block support (OLB)) would generate the smallest physical demands, the “intermediate” versions (with single-limb and wall support (Tree) and bilateral-limb and chair support (OLB)) would generate intermediate demands, and the most “advanced” versions (with unilateral-limb support alone (Tree and OLB)) would generate the greatest physical demands across all lower extremity (LE) joints and planes of motion. Our *a priori *hypotheses are based on the accumulative experience of the research team, which included a geriatrician, biomechanist, yoga instructor with extensive clinical-trial and senior-student training experience, and a physical therapist [12].

## 2. Subjects and Methods

### 2.1. Subjects

Participants in this study were part of the Yoga Empowers Senior Study (YESS) [12]. The YESS study sample consisted of 6 men and 14 women, aged 70.7 years (±3.8 years). Mailing lists, physician referrals, flyers, and newspaper advertisements were used to recruit the participants. Initial eligibility (e.g., age) was evaluated by phone, and then additional inclusion and exclusion criteria were assessed in person. The following were exclusions: active angina; uncontrolled hypertension (SBP > 160 or/and DBP > 90); high resting heart rate (greater than 90) or respiratory rate (greater than 24); unstable asthma or exacerbated chronic obstructive pulmonary disease; cervical spine instability or other significant neck injury; rheumatoid arthritis; unstable ankle, knee, hip, shoulder, elbow, or wrist joints; hemiparesis or paraparesis; movement disorders; peripheral neuropathy; stroke with residual deficit; severe vision or hearing problems; walker or wheelchair use; not able to attend in-person classes; has not had checkup by regular provider within 12 months (if not taking any prescription medications) or in the past 6 months (if any regular medicines taken). Participants also had to execute the following safety tests stably and independently: transition from standing to recumbent on the floor and reverse; lift both arms to shoulder level; stand with feet side-by-side for 30 seconds; stand with feet hip-width apart for 60 seconds. 

### 2.2. Study Design

The YESS study was a single armed pre-post intervention study of 2 specified series of Hatha yoga postures, conducted in ambulatory, community-dwelling adults aged 65 years or greater. The Yoga intervention was delivered 2 days per week, 1 hour per session, for 32 weeks. We used Hatha yoga, which teaches *asanas* (postures) and *pranayama *(breathing). The intervention consisted of 2 sets of postures, Series I and Series II, designed to be progressive (i.e., to advance in difficulty) and to train major muscle groups that are integral to conducting activities of daily living. These included the shoulder/upper extremity (necessary for reaching and for carrying light loads); trunk stabilizers (for balance); hip/lower extremity (for static and dynamic balance, locomotion, and transfers (e.g., sit to stand)). We used modified versions of standard *asanas*, tailored in a manner that we believed to be suitable for independent, ambulatory seniors. Specific postures were selected by the research team based upon our combined knowledge of yoga, biomechanics, physical therapy, and movement science. Reproducibility was also a specific goal; we therefore modeled the YESS program after the Ashtanga School, which uses a standard set of opening and closing postures, and a variable middle series which progresses in difficulty. Similarly, YESS used opening and closing sequences and 2 ordered, progressive middle sequences, termed Series I (first half of the study) and Series II (second half of the study). Using this standardized approach makes the intervention transparent to both the research and the teaching communities. 

This paper focuses on the biomechanical analysis of 2 poses, Tree (Vriksasana) and OLB (Utthita Hasta Padangusthasana) (see the following for description of biomechanical data collection and analysis). Three versions of each of these poses were instructed across the 2 series of the YESS intervention. The poses were “progressed” in difficulty by reducing the limb support from a bilateral stance (introductory versions) to single-limb stance (advanced versions) and by reducing or removing additional supporting structures (wall, chair, blocks). By the end of the study, the participants were practiced at performing all 3 versions of each pose. The object of the current analysis was to examine the biomechanics of the 3 progressive versions of each pose; therefore, we used biomechanical data from the final biomechanics study visit, during which biomechanics data on all 3 versions of each pose were obtained.

**Figure 1:**
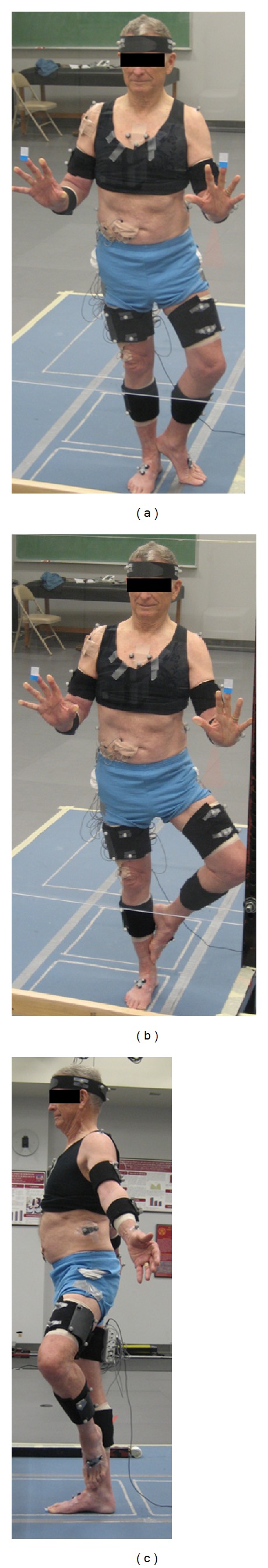
The variations of Tree pose, performed while the participant is instrumented for biomechanical analysis. (a) TreeWF: Tree pose with hands on wall and contralateral foot on the ground; (b) TreeW: Tree pose with hands on wall; (c) Tree: free-standing, single-support Tree pose.

The introductory version of Tree pose consisted of standing on the dominant limb (the limb with which one would kick a ball; we refer to this as the *stance* limb) and placing the contralateral foot on the floor, with toes and forefoot in contact with the floor and the heel resting just above the contralateral ankle. The participant's hands lightly touched a wall (TreeWF, Figure 1(a)). In the intermediate version of Tree, participants placed their contralateral foot on the medial aspect of the support-limb shank, just above the ankle (Figure 1(b)) and again used the wall for support (TreeW). The advanced version of Tree approached the classic posture; it was the same as version 2, but the wall support was not used (in the classical version of Tree, the contralateral foot is placed on the mid-to upper thigh) (Tree, Figure 1(c)).

**Figure 2:**
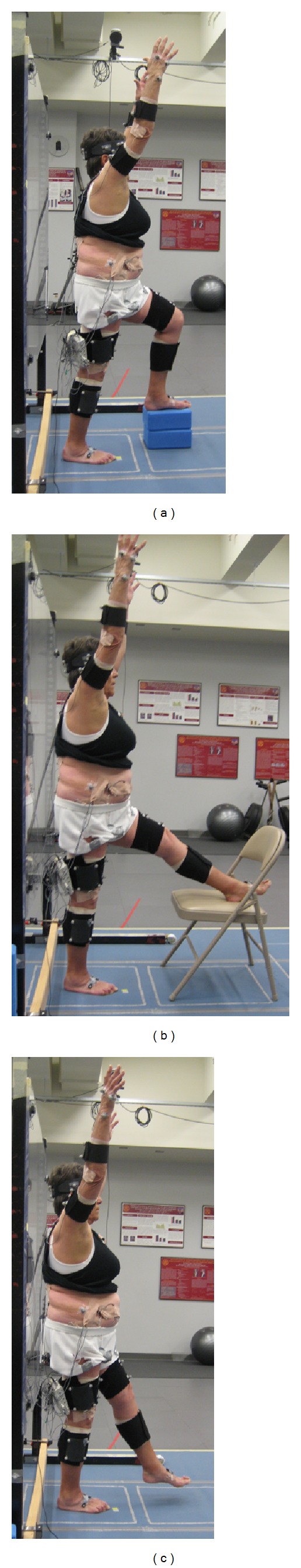
The variations of One-Leg Balance (OLB) pose, performed while the participant is instrumented for biomechanical analysis. (a) OLBb: double-support OLB pose with contralateral foot on the yoga blocks and knee flexed; (b) OLBc: double-support OLB pose with contralateral foot on a chair and knee extended; (c) OLB: free-standing, single-support OLB pose.

OLB was also a single-limb-stance pose in which the participants stood on their dominant limb. The introductory OLB variation included the use of 2 or 3 standard yoga blocks (OLBb; block height = 20 or 30 cm depending on participant height). Here the subjects placed their contralateral foot on the blocks with their knee flexed to 90° (Figure 2(a)). The intermediate version of OLB included the use of a chair (OLBc). Here, the participants flexed their contralateral hip and rested their extended limb on the seat of a standard folding chair (seat height = 44.5 cm; Figure 2(b)). The advanced version of OLB was a free standing pose with the contralateral limb lifted anteriorly with a flexed hip (OLB). They also lifted their arms above their head by flexing their shoulders to approximately 180° (Figure 2(c)). 

### 2.3. Biomechanical Assessments

Biomechanical analysis was performed at the USC Musculoskeletal Biomechanics Research Laboratory using standard techniques [13]. Whole-body kinematic data were collected using an eleven-camera motion capture system at 60 Hz (Qualisys Tracking System with Oqus 5 cameras; Qualisys, Gothenburg, Sweden). Reflective markers were placed on a head band and over the following anatomical landmarks of the lower and upper extremities bilaterally: first and fifth metatarsal heads, malleoli, femoral epicondyles, greater trochanters, acromions, greater tubercles, humeral epicondyles, radial and ulnar styloid processes, and third metacarpal heads. Markers were also attached to the spinous process of the 7th cervical vertebrae (C7), jugular notch, L5/S1, bilateral iliac crests, and bilateral posterior superior iliac spines, in order to define the trunk and pelvis. Based on these markers, a total of 15 body segments were modeled, including the upper arms, forearms, hands, head, trunk, pelvis, thighs, shanks, and feet. Noncollinear tracking marker plates were placed on each of these segments to track segmental position during the poses, using previously documented procedures [14, 15].

Once instrumented, the subjects performed the pose sequences, while guided by their instructor. The sequence of the poses was the same as when it was carried out in the regular yoga classes. A firm but portable clear plexiglass wall, which permitted capture of the markers, was positioned for wall support in the lab visits. For each pose, the participant was instructed to begin in a starting position, move smoothly into the pose, hold the pose while taking one full breath, and then return back to the original position. Simultaneously, the instructor also performed each pose in order to provide visual cueing. Once the participant had moved into the pose position, the instructor provided a verbal cue to the research associate to initiate the 3-second data collection. Two successful trials of each pose version were collected, and all 3 seconds of each pose were used for the analyses. 

The subjects also completed 2 successful walking trials at their self-selected “comfortable speed.” The walking trials provided a reference condition [16–18]. That is, walking is a well-studied, stereotypical activity and the JMOFs measured during comfortable-pace walking could be compared to the JMOF's generated by the 2 yoga poses.

GRFs were measured from a force platform at 1560 Hz (AMTI, Watertown, MA). Qualisys Track Manager Software (Qualisys, Gothenburg, Sweden) and Visual 3D (C-motion, Rockville, MD) were used to process the raw coordinate data and compute segmental kinematics and kinetics. Trajectory data was filtered with a fourth-order zero lag Butterworth 12 Hz low-pass filter. In Visual 3D, the head was modeled as a sphere, the torso and pelvis as cylinders, and the upper and lower extremity segments as frusta of cones. The local coordinate systems of body segments were derived from the standing calibration trial. Joint kinematics were computed based upon Euler angles with the following order of rotations: flexion/extension, abduction/adduction, internal/external rotation. The principle moments of inertia were determined from the subject's total body weight, segment geometry, and anthropometric data. Using standard inverse dynamics techniques, along with the International Society of Biomechanics recommended coordinate systems, net JMOFs in the sagittal and frontal planes, for the ankle, knee, and hip, were calculated from the inertial properties, segmental kinematics, and GRFs [19, 20]. JMOFs were normalized to each subject's bodyweight in kg. Additionally, a support moment, calculated as the sum of the ankle, knee, and hip sagittal plane JMOFs, was determined for each pose [17, 21].These instrumentation and data-processing techniques have previously been used in our laboratory to assess exercise performance with high reliability (Cronbach's alpha = 0.98; [22]). 

Surface electromyographic (EMG) signals of the lower gluteus medius, hamstrings, vastus lateralis, and gastrocnemius muscles were collected on the subjects' dominant limb at 1560 Hz using active surface electrodes (Motion Lab Systems, Baton Rouge, LA). Standard procedures, including preparation of the skin and electrode placement, were employed [23]. The obtained EMG signals were amplified (×1000), notch filtered at 60 Hz, and band-pass filtered at 20–500 Hz. A root mean square smoothing algorithm [24] with a 75 ms constant window was used to smooth the EMG data over the 3-second data collection period corresponding to the epoch of kinematic and kinetic data. EMG processing and smoothing were performed using MATLAB (MathWorks, Natick, MA). The study protocol was approved by the Institutional Review Boards at the University of Southern California (USC) and University of California at Los Angeles (UCLA), and all subjects provided their written consent to participate.

### 2.4. Statistical Analysis

The JMOFs of the stance limb ankle, knee, and hip joints, in the sagittal and frontal plane, were the primary dependent variables. These were averaged over the collected period and both repetitions. Secondary dependent variables included the EMG activity, which was also averaged across the 2 trials. Repeated measures ANOVA (1 group × 3 tasks) were used as omnibus tests to identify significant differences in the JMOFs and EMG results for each dependent variable within each pose group (Tree and OLB). Tukey's post hoc tests were used to examine the pairwise comparisons between tasks in each group when the ANOVA tests were significant. Additionally, Cohen's *d* effect sizes (small *d* = 0.2; medium *d* = 0.5; large *d* = 0.8) are reported for all statistically significant post hoc comparisons [25]. Statistical analysis was conducted via PASW Statistics 18 (IBM SPSS Statistics, Armonk, NY), and significance level was set at *P* < 0.05. 

## 3. Results

### 3.1. Self-Selected Walking JMOFs

The average peak JMOFs across the self-selected walking trials are presented in Table 1. These JMOFs generated during walking provide a metric against which the pose JMOFs can be compared.

### 3.2. Tree Support JMOF

**Figure 3:**
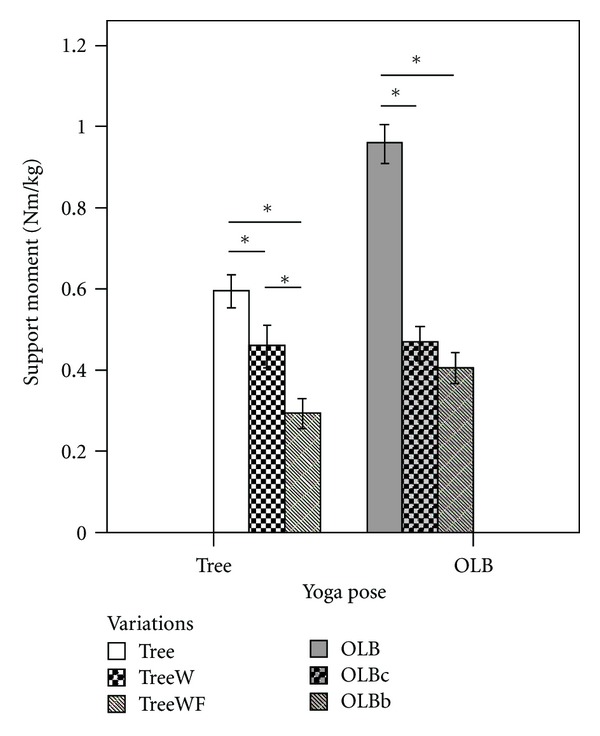
Magnitude of support JMOF during variations of Tree and OLB. The whiskers represent standard errors (*P < 0.05).

The repeated measures ANOVA test identified a significant difference in the support JMOF among the 3 Tree variations (*F*_2,38_ = 36.12; *P* < 0.001). Pairwise comparisons (post hoc analysis) revealed that the support JMOF during the classical Tree pose (the advanced version) was 30% (*P* = 0.001, *d* = 0.65) greater than Tree with wall support (TreeW, the intermediate version) and 103% greater than Tree with toes touching the floor and wall support (TreeWF, the introductory version) (*P* < 0.001, *d* = 1.76; Figure 3). Additionally, the TreeW support JMOF was 57% greater than TreeWF (*P* < 0.001, *d* = 0.83). 

#### 3.2.1. Tree Sagittal Plane JMOFs and EMG

**Figure 4:**
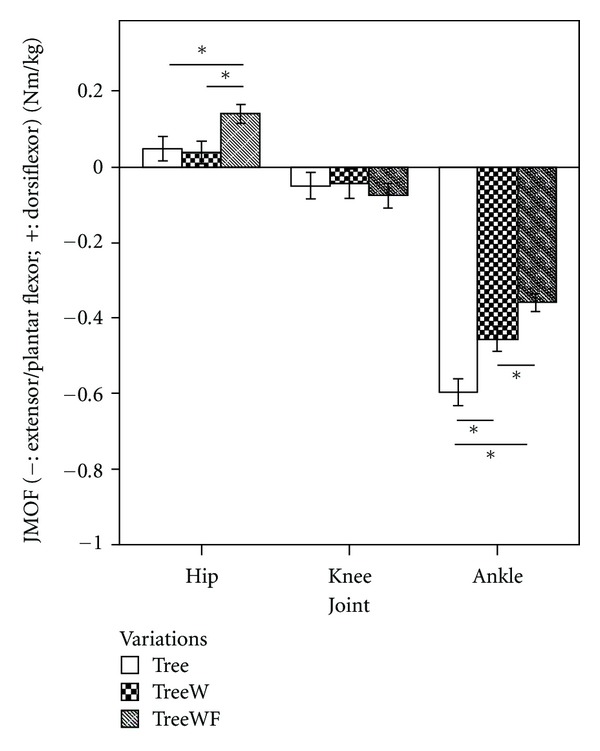
Mean JMOFs in the sagittal plane during Tree variations. The whiskers represent standard errors (*P < 0.05).

Figure 4 illustrates comparisons of the JMOF at the hip, knee, and ankle in the sagittal plane across Tree variations. Repeated measures ANOVA identified significant differences in hip flexor and ankle plantar flexor JMOFs (*F*_2,38_ = 13.36 and 29.40, resp.; *P* < 0.001), but not the *knee extensor *JMOF (*F*_2,38_ = 0.795; *P* = 0.46). Pairwise comparisons revealed that the average hip flexor JMOF generated during TreeWF was 188% greater than classical Tree (*P* < 0.001, *d* = 0.70) and 268% greater than TreeW (*P* < 0.001, *d* = 0.83). There was not a statistically significant difference between the 2 single-support Tree versions (Tree and TreeW; *P* = 0.88). Regarding the ankle plantar flexor JMOF, the pairwise comparison demonstrated that the traditional Tree induced a JMOF that was 31% greater than TreeW (*P* < 0.001, *d* = 0.92) and 66% greater than TreeWF (*P* < 0.001, *d* = 1.77); while TreeW was 27% greater than the introductory TreeWF (*P* = 0.01, *d* = 0.75). 

Surface EMG signals from the gastrocnemius muscles were used to support the sagittal-plane JMOFs at the ankle (Table 2). Significant differences were identified across the Tree versions in gastrocnemius activation level (*F*_2,38_ = 68.29; *P* < 0.001). Average gastrocnemius activation during the classical Tree was 123% greater than Tree with wall support (TreeW; *P* < 0.001, *d* = 1.29) and 324% greater than Tree with toes touching the floor and wall support (TreeWF; *P* < 0.001, *d* = 1.94). Gastrocnemius activation during TreeW was 90% greater than that during TreeWF (*P* = 0.052, *d* = 0.91).

#### 3.2.2. Tree Frontal Plane JMOFs and EMG

**Figure 5:**
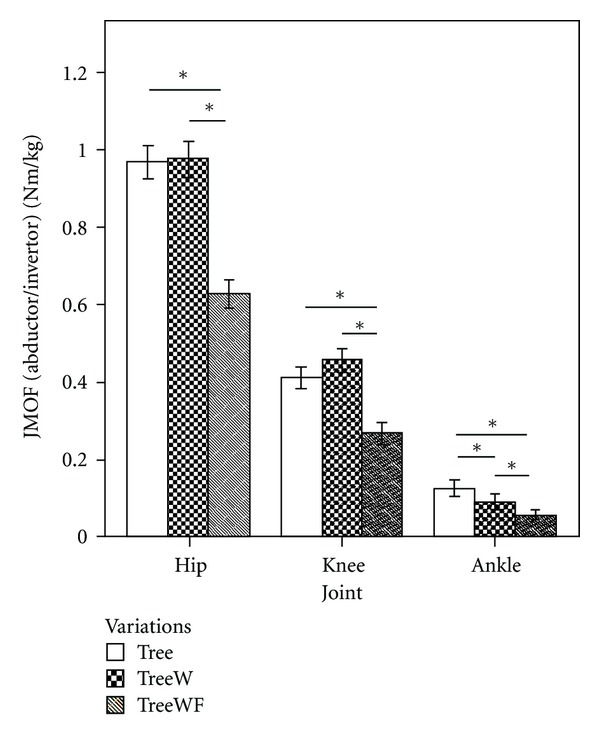
Mean JMOFs in the frontal plane during Tree variations. The whiskers represent standard errors (*P < 0.05).

In the frontal plane, the overall statistical analyses identified significant differences across the 3 Tree variations at each LE joint (*F*_2,38_ = 50.05, 36.44, and 19.03 for, hip, knee, and ankle, resp.; *P* < 0.001). Pairwise comparisons revealed that the 2 single-support Tree poses (Tree and TreeW) engendered similar hip abductor JMOFs (*P* = 0.98) which were approximately 55% greater than the JMOF engendered during the introductory version TreeWF (*P* < 0.001, *d* = 1.90). Complementing the frontal-plane JMOF findings, the classical Tree and TreeW poses generated higher average gluteus medius EMG signals (85% and 54%, resp.) than TreeWF. However, statistical significance in the EMG level was only found in the difference between the classical Tree and TreeWF (*P* = 0.007, *d* = 0.60), and only a trend was identified between TreeW and TreeWF (*P* = 0.12). Gluteus medius activation also did not differ between the traditional Tree and TreeW (*P* = 0.46). Similar to findings related to the hip joint, the classical Tree and TreeW produced knee abductor JMOFs which were 54% and 71% greater than the JMOF generated by the TreeWF, respectively (*P* < 0.001, *d* = 1.12 and 1.43; Figure 5). All 3 Tree variations produced ankle invertor JMOFs. The classical Tree ankle invertor JMOF was 40% greater than that generated during TreeW (*P* = 0.006, *d* = 0.39) and 118% greater than that generated during the introductory version, TreeWF (*P* < 0.001, *d* = 0.87). 

### 3.3. OLB Support JMOF

Similar to the Tree findings, the repeated measures ANOVA test identified that the support JMOF differed statistically among the 3 OLB variations (*F*_2,38_ = 97.00; *P* < 0.001). Pairwise comparisons revealed that the support JMOF during the classical OLB pose (the advanced version) was 106% greater than OLB with foot on a chair (OLBc, the intermediate version; *P* < 0.001, *d* = 2.48) and 137% greater than OLB with foot on blocks (OLBb, the introductory version; *P* < 0.001, *d* = 2.90). There was not a significant difference in support JMOF between OLBc and OLBb (*P* = 0.36). 

#### 3.3.1. OLB Sagittal Plane JMOFs and EMG

**Figure 6:**
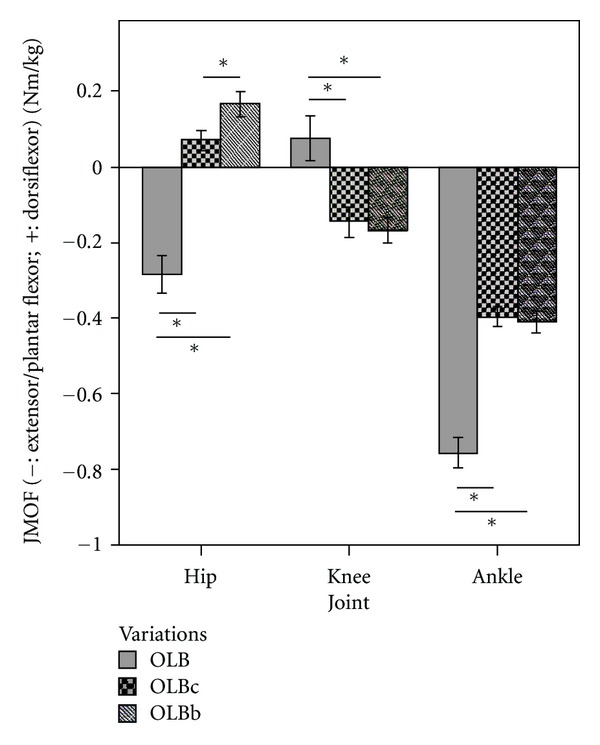
Mean JMOFs in the sagittal plane during OLB variations. The whiskers represent standard errors (*P < 0.05).

Comparisons of JMOFs at the hip, knee, and ankle in the sagittal plane are illustrated in Figure 6, and EMG data for the OLB versions are presented in Table 3. Repeated measures ANOVAs identified statistical differences among OLB variations in sagittal plane JMOFs at all 3 LE joints (*F*_2,38_ = 92.55, 36.10, and 54.69 for hip, knee, and ankle, resp.; *P* < 0.001). At the hip, an extensor JMOF was generated during the traditional OLB; whereas a flexor JMOF was generated during the OLBb which was 135% greater than the flexor JMOF generated during the OLBc (*P* = 0.02, *d* = 0.72). At the knee, the classical OLB generated a flexor JMOF, whereas similar extensor JMOFs were generated with the 2 double-support OLB variations (OLBb and OLBc) (*P* = 0.74). At the ankle, pairwise comparisons revealed that the plantar flexor JMOF associated with the traditional OLB was 85% greater than OLBb (*P* < 0.001; *d* = 2.17) and 91% greater than OLBc (*P* < 0.001; *d* = 2.34). The plantar flexor JMOF did not differ between OLBb and OLBc variations (*P* = 0.94). These ankle plantar-flexor results were supported by the gastrocnemius EMG data. The classical OLB pose induced significantly greater gastrocnemius muscle activation than the intermediate version of OLB with foot on a chair (OLBc; 138%; *P* < 0.001, *d* = 1.61) and the introductory version of OLB with foot on blocks (OLBb; 185%; *P* < 0.001, *d* = 1.74). No significant EMG difference was found between the OLBc and OLBb (*P* = 0.605). 

#### 3.3.2. OLB Frontal Plane JMOFs and EMG

**Figure 7:**
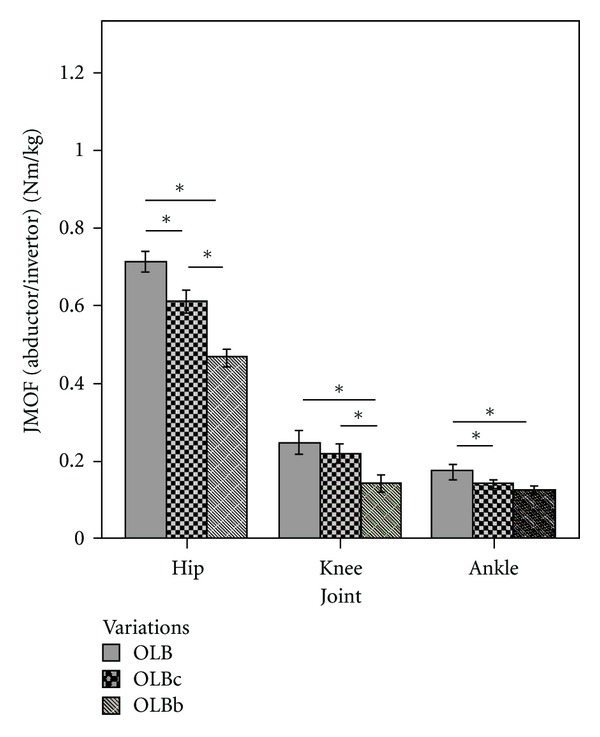
Mean JMOFs in the frontal plane during OLB variations. The whiskers represent standard errors (*P < 0.05).

In the frontal plane, the JMOFs generated at each LE joint differed statistically among the 3 OLB variations (*F*_2,38_ = 80.66, 21.92, and 11.94 for hip, knee, and ankle, resp.; *P* < 0.001). The traditional OLB induced a hip abductor JMOF which was 17% greater than that induced during the intermediate version, OLBc (*P* < 0.001, *d* = 0.82), and 53% greater than that produced by the introductory version, OLBb (*P* < 0.001, *d* = 2.24) (Figure 7). A higher gluteus medius activation was also induced by the traditional, single-support OLB pose compared to OLBc (43%, *P* = 0.003, *d* = 0.53) and OLBb (129%, *P* < 0.001, *d* = 1.12). Complementing the significant differences (31%) found in the hip abductor JMOF between the 2 double-support OLB variations (OLBc and OLBb; *P* < 0.001, *d* = 1.22), gluteus medius activation during OLBc was 60% greater than that during OLBb (*P* = 0.009, *d* = 0.66). At the knee, the pairwise comparison revealed that the advanced OLB and intermediate OLBc poses produced abductor JMOFs which were 76% (*d* = 0.91) and 55% (*d* = 0.75) greater than that produced during the introductory version, OLBb (*P* < 0.001). There was not a difference in knee abductor JMOF between traditional OLB and OLBc (*P* = 0.21). At the ankle, the invertor JMOF produced during the traditional OLB pose was 23% greater than that produced during OLBc (*P* = 0.007, *d* = 0.46) and 38% greater than that produced during OLBb (*P* < 0.001, *d* = 0.68). No difference was observed between OLBb and OLBc (*P* = 0.27). 

## 4. Discussion

In this study, we quantified the JMOFs and average EMG activity associated with 3 versions of yoga poses that are traditionally done standing on one leg: Tree and One-Leg Balance. The 3 versions of each pose progressively increased the difficulty level of performing each version from introductory, through intermediate, to advanced. By conducting biomechanical tests during the performance of each version of Tree and One-Leg Balance, we formally evaluated whether the poses were indeed becoming more physically demanding as hypothesized.

Our findings, which were not always intuitive, demonstrated that, within each pose, the different variations engendered significantly different JMOFs and EMG activity, in some but not all joints, planes of motion, and muscle groups. Thus, our general hypothesis that the introductory poses would generate the smallest physical demands, the intermediate versions would generate intermediate demands, and the most advanced versions would generate the greatest physical demands, in a uniform fashion that is across all LE joints and planes of motion, was not upheld. Moreover, we were surprised to find that within a given pose type (e.g., One-Leg Balance) some pose variations generated JMOFs in opposite directions (i.e., hip flexor versus hip extensor) and thus were likely to target antagonistic muscle groups and opposing articular structures. In the following sections, we discuss the meaning and implications of the biomechanical findings for the Tree and One-Leg Balance poses, in turn.

### 4.1. Tree

For the support moment, our findings supported our hypothesis that the classical Tree would generate the greatest support moment, followed by Tree with wall support and then Tree with toes on floor and wall support (i.e., Tree > TreeW > TreeWF). The support moment is calculated as the sum of the individual extensor moments at the hip, knee, and ankle; consequently, this comprehensive measure is considered a good indicator of the *overall* demand on the lower extremity [16, 21, 26, 27]. Our findings demonstrated that there is a nonlinear relationship across the 3 variations with a large increase (57%) from TreeWF to TreeW and only a moderate increase (30%) from TreeW to Tree. Therefore, based on these analyses, the degree of difficulty change between the introductory and intermediate versions is much greater than the increment in difficulty between the intermediate and advanced versions. These results suggest that more time may be needed practicing the introductory version (TreeWF) before transitioning to the intermediate version (TreeW); whereas less time may be needed in the progression from the intermediate version to the advanced, classical Tree version. 

In contrast to the support moment and ankle JMOF findings, our Tree pose findings were not intuitive for the hip and knee JMOFs—in other words, the hypothesized relations that the classical Tree would generate the greatest JMOFs, followed by Tree with wall support, and then Tree with toes on floor and wall support did not hold. For example, in the frontal plane the Tree and TreeW produced almost identical hip abductor JMOFs which were approximately 55% greater than TreeWF (a large effect size) and 12% greater than the average peak JMOF produced during the walking trials. The EMG findings for the gluteus medius (a primary hip abductor) were consistent with the JMOF results. 

Hip abductor strength is important because it is associated with balance performance [28–30] and fall risk [31] in seniors. Consequently, exercise programs designed to improve balance and reduce fall risk should include activities which target the hip abductors. We had hypothesized that using a wall for balance support (i.e., TreeW) would reduce the frontal-plane demands and gluteus medius activity associated with the advanced pose (Tree) by allowing the participants to shift their center of mass location closer to their hip joint center. This, however, was not supported by the biomechanical evidence. Clinically, these findings are important and imply that instructors can increase the demands to the hip abductors early in a yoga program, even in participants that have relatively poor balance capabilities and need to use a wall for support. 

In the sagittal plane at the hip, surprisingly, it was the introductory TreeWF variation that generated the greatest flexor JMOF (a modest-to-large effect size), whereas no difference was found between Tree and TreeW. Hip flexor performance is positively associated with stride length and walking speed in seniors [16, 32, 33], and DiBenedetto and colleagues [34] reported that both of these parameters increased in older adults following an 8-week Iyengar Hatha yoga program. Clinically, our findings suggest that the hip flexors (e.g., psoas major and iliacus) can be targeted early in a yoga intervention program using the introductory TreeWF pose, even when participants with limited balance capabilities need to use a wall and their contralateral limb for support.

At the knee, all 3 Tree poses produced a similar extensor JMOF in the sagittal plane. This JMOF, however, was small and only 8.8% of the average peak extensor moment generated during self-selected walking. These findings are not surprising because the extended knee position associated with all 3 versions of the Tree results in a GRF projection which is close to the knee joint axis of rotation. Contrastingly, when the knee is flexed during the loading phase of gait, the GRF moves progressively posterior to the knee joint and an appreciable knee extensor JMOF is generated. Thus, none of the Tree versions examined, in and of themselves, is likely to improve knee extensor strength or endurance.

In the frontal plane, Tree and TreeW produced similar knee abductor JMOFs which were 54–71% greater than that generated during TreeWF and were greater (8–20%) than the average peak knee abductor JMOF produced during walking at self-selected speed. These findings have important implications for participants with knee pathology because high and/or sustained knee abductor JMOFs will increase the loading of the MCL and compressional loading across the lateral condyles and lateral patellofemoral surfaces. These loading characteristics are associated with OA [10, 11, 35], and joint pain [36, 37]; thus, the intermediate and advanced tree poses could exacerbate preexisting conditions. Importantly, and in contrast to commonly-held conceptions, the use of a wall for support during Tree posing will not diminish the frontal plane JMOF or offer protection for the knee joint.

### 4.2. OLB

Similar to the Tree results, most of the OLB JMOF findings were in contrast to our stated hypotheses that the traditional One-Leg Balance pose (OLB) would induce greater JMOFs than the intermediate One-Leg Balance pose with chair (OLBc), which would induce a JMOF which was greater than the One-Leg Balance pose with blocks (OLBb) (i.e., OLB > OLBc > OLBb). For example, the support moment generated during the advanced OLB pose was 106–137% greater than OLBc and OLBb (a large effect size); however, the average support moment did not differ between OLBb and OLBc. These findings suggest that, in terms of overall LE demand, a considerable increase in effort will be required as participants advance from the introductory and intermediate OLB poses to the advanced OLB pose.

At the hip, the abductor JMOF results were the only OLB findings that supported our stated hypothesis. The hip abductor JMOF for the OLB was 17% greater than OLBc and 53% greater than OLBb (large effect sizes). Thus, there appears to be a nearly linear progression in demand across the 3 OLB variations, suggesting that a similar amount of practice is likely to be necessary in order to progress a participant from OLBb to OLBc and from OLBc to OLB. In relation to the JMOF produced during self-selected walking, OLB produced a sustained hip abductor JMOF that was 82% of the average peak JMOF generated during the walking trials. Thus, the OLB pose sequence, like the Tree pose sequence, may be a good addition to yoga programs designed to increase hip abductor strength, improve balance, and reduce fall risk in seniors.

In the sagittal plane, the hip JMOF results were quite interesting. While the classical OLB generated a hip *extensor* JMOF that was 35% of the average peak extensor JMOF generated during gait, OLBc and OLBb generated hip *flexor* JMOFs which were 13% and 31% of the average peak *flexor* JMOF generated during gait. Thus, these 3 variations target antagonistic muscle groups and opposing joint articular structures. Nonetheless, because these JMOFs (both flexor and extensor) were well below those generated during self-selected walking, their inclusion in yoga programs designed to increase walking performance via improvements in hip flexor/extensor strength is not supported.

Similar results were found in the sagittal plane JMOFs at the knee. Whereas the classical OLB pose generated a knee *flexor* JMOF, the intermediate OLBc and the introductory OLBb poses generated similar *extensor* JMOFs. Both extensor and flexor JMOFs, however, were well below those generated during the gait trials—76% and 74% less, respectively. Like the Tree pose variations, the small sagittal plane JMOFs at the knee are likely the result of an almost vertical alignment of the thigh and shank, and small knee flexion/extension angles which position the projection of the GRF close to the knee joint center. Consequently, these poses are not likely to have a large influence on knee extensor/flexor strength or endurance. Interestingly, hamstring EMG activity increased dramatically when subject performed the classic OLB pose, in concert with the knee flexor JMOF; however, the EMG activity level of the vastus lateralis only decreased slightly between the intermediate to advanced versions. Thus it appears that the participants used a cocontraction strategy at the knee during performance of the advanced, classic, OLB pose, potentially stiffening the joint and increasing stability. This cocontraction strategy, however, will increase the loading across the tibiofemoral condyles and thus, could exacerbate existing OA symptoms (e.g., pain).

In the frontal plane at the knee, OLB and OLBc generated JMOFs which were 76% and 55% greater than that of OLBb, respectively. Both of these differences demonstrated large effect sizes; however, all 3 variations engendered knee abductor JMOFs that were appreciably less than the average peak JMOF engendered during gait. Thus, these pose variations are not likely to be riskier than walking programs in aggravating preexisting knee pathologies. 

A note of caution, however, when comparing the JMOFs generated during yoga poses with those generated during walking, it is important to consider that walking is a cyclic activity in which the JMOFs increase and decrease during a gait cycle. Thus, we calculated and recorded the *peak* JMOFs, across the hip, knee, and ankle, which were produced during the walking trials. Contrastingly, during yoga practice the participants statically held their poses “for a full breath” before returning to a starting position, and we calculated the average JMOFs engendered during the middle 3 seconds of each pose. Thus, a fair comparison between the JMOFs engendered during yoga and walking should take into consideration that the peak JMOFs reported during walking only occur for an instant in time, whereas the average JMOFs produced during each yoga pose persist for 5-6 seconds. Consequently, although the peak JMOFs produced during dynamic activities such as walking may be greater than those generated during the yoga poses, the overall muscular stimulation and extended joint loading, that occurs during yoga posing, may be greater than that produced during walking or other dynamic activities (e.g., resistance exercise).

At the ankle, OLB generated an ankle plantar flexor JMOF that was approximately 88% greater than the JMOF generated during OLBb and OLBc (large effect size) and 57% of the average peak JMOF generated during gait. Plantar flexor strength and performance are associated with balance and postural control [38, 39], walking performance [40], and fall risk [41–43] in seniors. Our findings suggest, however, that it is not until participants are able to safely perform the advanced version of the OLB pose, that they will appreciably load their plantar flexor muscles. This is also supported by the results of muscle activity level in ankle plantar flexor. EMG level of gastrocnemius induced by the advanced version was approximately 123% greater than that induced by the other two versions.

In the frontal plane at the ankle, OLB produced an invertor JMOF that was approximately 30% greater than the JMOF generated during OLBb and OLBc (small to modest effect size) and similar to the average peak JMOF generated during gait. Ankle invertor strength is important for balance and safe ambulation, and it is correlated with performance in the timed up-and-go test in seniors [38]. Because this test requires a combination of strength, balance, and agility, it is often considered a “comprehensive” measure of ambulatory proficiency [44–46]. Our findings suggest that performance of the classical OLB pose will target the ankle invertors (e.g., tibialis anterior and posterior) and thus may ultimately improve dynamic balance and ambulation proficiency.

## 5. Conclusions

This is the first study to quantify the physical demands of yoga pose variations, using biomechanical methodologies. This paper is informative and provides evidence that can be used by instructors, clinicians, and therapists to help select pose variations which are appropriately tailored to the experience, physical capabilities, and injury history of participants. Although we only examined 2 poses and 3 variations of each, our biomechanical analysis was comprehensive and included the kinetic examination of 3 LE joints across 2 planes of motion, and the study of 4 functionally important muscle groups. Future studies are needed to examine additional poses which are prevalent in older-adult yoga programs and their common variations.

Importantly, our findings were not always intuitive and suggest that common, long-held, conceptions about the demands placed on the body by poses should be investigated experimentally. For example, pose variations which have long been considered introductory may actually induce approximate or even higher demands at some joints and planes of motion, than pose variations considered advanced. Similarly, we were surprised to find that some pose variations (e.g., OLBb and OLBc) induced JMOFs which were in the opposite direction of those generated during the classical variation (OLB). Finally, we demonstrated that the use of props, such as a wall, to reduce contraindicated joint loading (e.g., knee frontal plane JMOF) may have little or no effect. While these findings are informative, evidenced-based yoga programs designed using this type of biomechanical information will ultimately have to be tested experimentally, within randomized controlled trials, to determine their influence on program safety, participant retention, physical-performance efficacy, and quality of life in seniors.

## Figures and Tables

**Table 1:** Lower extremity peak net joint moments of force during the stance phase of gait at a self-selected walking speed.

Moments (Nm/kg)	Hip	Knee	Ankle
*Sagittal plane*			
Extensor	0.81 ± 0.06	0.63 ± 0.04	1.33 ± 0.04^‡^
Flexor	0.54 ± 0.03	0.30 ± 0.03	0.29 ± 0.06^†^
*Frontal plane*			
Abductor	0.87 ± 0.04	0.38 ± 0.03	0.09 ± 0.01
Adductor	0.08 ± 0.01	0.09 ± 0.01	0.19 ± 0.02

Mean ± standard error.

^‡^Plantar flexor moment.

^†^Dorsiflexor moment.

**Table 2:** Average EMG activity of lower extremity muscle groups during Tree poses.

EMG (mV)	TreeWF	TreeW	Tree
Gluteus Medius	17.35 ± 3.26	26.64 ± 4.46	32.15 ± 7.13
Hamstrings	11.96 ± 2.63	24.70 ± 7.36	48.05 ± 10.60
Vastus lateralis	62.22 ± 15.02	77.37 ± 20.46	101.47 ± 23.71
Gastrocnemius	24.33 ± 3.75	46.21 ± 6.63	103.12 ± 12.31

Mean ± standard error.

**Table 3:** Average EMG activity of lower extremity muscle groups during OLB poses.

EMG (mV)	OLBb	OLBc	OLB
Gluteus medius	19.34 ± 2.95	30.95 ± 4.67	44.28 ± 6.38
Hamstrings	21.49 ± 8.29	29.47 ± 6.95	94.78 ± 13.55
Vastus lateralis	78.20 ± 15.03	106.89 ± 20.40	86.92 ± 12.50
Gastrocnemius	41.37 ± 6.32	49.48 ± 5.16	117.72 ± 12.33

Mean ± standard error.
